# Methylcellulose Hydrogel with *Melissa officinalis* Essential Oil as a Potential Treatment for Oral Candidiasis

**DOI:** 10.3390/microorganisms8020215

**Published:** 2020-02-06

**Authors:** Elisa Serra, Fabien Saubade, Cosimo Ligorio, Kathryn Whitehead, Alastair Sloan, David W. Williams, Araida Hidalgo-Bastida, Joanna Verran, Sladjana Malic

**Affiliations:** 1 Microbiology at Interfaces, Faculty of Science and Engineering, Manchester Metropolitan University, Manchester M1 5GD, UK; elisaser@hotmail.it (E.S.); fibus@hotmail.fr (F.S.); k.a.whitehead@mmu.ac.uk (K.W.); a.hidalgo@mmu.ac.uk (A.H.-B.); j.verran@mmu.ac.uk (J.V.); 2 Department of Materials, School of Natural Sciences, The University of Manchester, Manchester M13 9PL, UK; cosimo.ligorio@postgrad.manchester.ac.uk; 3 School of Dentistry, School of Dentistry, Cardiff University, Cardiff CF14 4XY, UK; alastair.sloan@unimelb.edu.au (A.S.); WilliamsDD@cardiff.ac.uk (D.W.W.)

**Keywords:** hydrogel, essential oil, oral candidiasis, rheology, antifungal activity, melissa oil

## Abstract

*Candida* spp. are the most prevalent fungi of the human microbiota and are opportunistic pathogens that can cause oral candidiasis. Management of such infections is limited due to the low number of antifungal drugs available, their relatively high toxicity and the emergence of antifungal resistance. Therefore, much interest in the antimicrobial potential of natural compounds has recently been evident. The use of hydrogels in the delivery of biocides has been explored due to their biocompatibility, ease with drug encapsulation, and due to their potential to confer mechanical and structural properties similar to biological tissue. Methylcellulose hydrogels (10% (*w*/*v*)) with 1% (*v*/*v*) and 2% (*v*/*v*) *Melissa officinalis* oil were synthesised. The rheological properties and gelation time of the hydrogels were evaluated. Antimicrobial action, the antifungal potential and ability to displace *Candida* were determined. Rheological tests revealed that the hydrogel jellified in three minutes at 37 °C. Loaded hydrogels successfully inhibited *Candida* *albicans* growth as evident by zone of inhibition and time-kill assays. A significant reduction in retained *C. albicans* was demonstrated with the hydrogel at 2% *Melissa officinalis* concentration. This work demonstrated that an essential oil-loaded hydrogel had the potential to provide a novel antimicrobial therapy for the treatment of oral candidiasis.

## 1. Introduction

*Candida albicans* is a commensal fungus found in 30–50% of healthy humans [1]. However, changes to the oral environment can lead to one of the most common human fungal infection, oral candidiasis. Treatments for oral candidiasis often involves administration of topical or systemic antifungals, and denture cleansers in case of denture stomatitis [2].

Compared to antibiotics, commercially available antifungals are limited and development without toxicity is complicated due to the eukaryotic nature of *Candida* [2]. The rise of resistance of *Candida* to clinically used antifungals has led to great interest in the antifungal properties of natural compounds [3]. 

Essential oils are products of aromatic plants and mainly composed of terpenes and terpenoids and other molecules [4]. Being hydrophobic, they interact with the cell membrane changing its permeability and causing leaching of intracellular components (e.g., radicals, proteins, calcium ions) and the inactivation of enzymatic mechanisms [5]. *Melissa officinalis* is a plant already used to give fragrance to food and beverage products [6]. Furthermore, it has been utilised as a medical plant for different therapeutic effects (e.g., energizer, anticonvulsant, tranquilizer, and to aid digestion) [7]. Besides these functions, it has been demonstrated to have both antimicrobial and antioxidant properties against periodontal pathogens [8,9] and *Candida* both in planktonic and biofilm form [10,11]. 

Hydrogels are 3D-networks obtained from natural and/or synthetic polymers that once cross-linked form an insoluble structure [12]. The property of hydrogels is their ability to absorb and retain a significant amount of water (>90%) without dissolving. Swollen hydrogels have some properties in common with living tissues (e.g., consistency, low interfacial tension, water content) and biocompatibility [13]. Therefore, they have been widely used in tissue engineering and regenerative medicine, mainly as drug and/or cell delivery systems, as pre-formed or injectable cell scaffolds [12]. 

Methylcellulose is widely used in food packaging. Formulations of methylcellulose films containing ginja cherry extract [14], olive leaf extract [15], clove bud (*Syzygium aromaticum*) and oregano (*Origanum vulgare*) essential oils [16] that demonstrate antimicrobial properties have been synthesised. The use of methylcellulose as a drug delivery system, has potential since it is a bioadhesive polymer that shows adhesion to mucosal surfaces [17]. This characteristic has the potential to prolong the retention of the drug delivery system at the site of clinical application (oral mucosa and/or vaginal applications) [18]. 

The aim of this study was to develop a methylcellulose hydrogel in combination with *Melissa officinalis* essential oil as treatment for oral candidiasis.

## 2. Materials and Methods

### 2.1. Hydrogel Preparation

Methylcellulose (M7140, Sigma-Aldrich, Gillingham, UK) was used to prepare hydrogels (10% (*w*/*v*)) containing 1% and 2% (*v*/*v*) *Melissa officinalis* (Essential Oils Direct Ltd., Oldham, UK) essential oil. *Melissa officinalis* (Essential Oils Direct Ltd., Oldham, UK) was used as in a previous study by Serra et al. [11]. Sterile distilled water, with or without 1% or 2% (*v*/*v*) *Melissa officinalis* and Tween 80 in equal amounts, was held at 80 °C for 10 min. Methylcellulose (10% (*w*/*v*)) was then gently added into the solvent. Once the powder was fully wet, the temperature was reduced by placing the solution on ice to form a clear viscous solution which was vortexed for 30 s until the powder was solubilised and then incubated overnight at 4 °C shaking at 200 rpm. Prior to use, the solution was vortexed for 1 min and equilibrated at room temperature for 1h to allow air bubble dispersion. The equilibrated solution was transferred to a Petri dish and incubated at 37 °C until a stable hydrogel was formed (within 30 min). Discs of the hydrogel were cut with a sterile cork-borer at different sizes (0.75 mm, 10 mm or 11.25 mm in diameter). 

### 2.2. Rheology

The rheological characterisation was performed on a Discovery Hybrid Rheometer DHR-2, (TA Instruments, DE) equipped with a 20 mm diameter plate and a solvent trap to minimise evaporation. Hydrogel solution (180 µL) was placed on the rheometer and the rheometer head was lowered to the desired gap height of 500 µm. Hydrogel gelation was studied under time sweep at 37 °C, while hydrogel viscoelasticity was analysed under strain and frequency sweep after keeping the hydrogel solution at 37 °C for eight minutes, with a gap size of 500 µm. Strain, frequency and time sweeps were carried out respectively using the following parameters: Equilibrium time of 8, 8 and 0 min; Frequency of 1 Hz, 0.01–100 Hz, 1 Hz; % Strain 0.1–100%, 1%, 1% and running time of 10 min for time sweep tests (*n* = 3) [19].

### 2.3. Melissa Officinalis Composition

Prior to evaluating the release of *Melissa officinalis* from the methylcellulose hydrogels, the composition of the essential oil was analysed by gas chromatography (Agilent Technologies 7890BUK) coupled to a mass spectrometer (Agilent Technologies 5977B, UK). The gas chromatograph was equipped with a capillary column Agilent J&W HP-5ms (30 m × 0.25 mm, 0.25 µm film thickness) (Agilent Technologies, UK). The method was adapted from that previously reported by Rajkowska et al. (2016) [20].

The initial temperature was set to 50 °C and held for 3 min, followed by a linear increase up to 300 °C (40 °C/min) and held for 2 min. A 5:1 split ratio with an injection volume of 2 μL was set. Inlet and transfer line temperatures were kept at 275 °C and 300 °C, respectively. Helium, CP grade, was used as a carrier gas at a flow rate of 1 mL/min. Quadrupole temperature was fixed to 150 °C. The mass spectra were obtained by scanning a range of masses from 45 to 550 atomic mass unit (AMU). The ion source temperature was 230 °C and the ionisation was obtained by electron impact at 70 eV. All samples were prepared in dichloromethane (DCM) and the composition of the oil was analysed by comparing the mass spectra with those of the computer library (NIST14 MS Search library).

Once the compounds were identified, abundance was determined using standard curves of each compound. Briefly, analytical standards of citronellol, citronellal, geraniol, and linalool (Sigma-Aldrich, UK) were used in double serial dilutions of each compound from 20 ppm to 2.5 ppm were prepared in DCM and analysed by GC-MS. The correlation between peak areas and concentrations was obtained and used to evaluate the abundance of each compound.

### 2.4. Melissa Officinalis Release

Ten percent (10% (*w*/*v*)) methylcellulose hydrogels containing 1% and 2% (*v*/*v*) *Melissa officinalis* essential oil were prepared. Once gellified at 37 °C, 1 g of hydrogel was cut with a 11.25 mm diameter sterile cork-borer and incubated in 20mL distilled water in a sterile glass universal at 37 °C under shaking conditions at 150 rpm (New Brunswick™ I26, Eppendorf UK Limited, Stevenage, UK). Two millilitres of liquid were collected at 1, 2, 3, 4, 5, 6, 7, 8, 24, 30 and 48 h and replaced by 2 mL of distilled water, to keep the volume constant throughout the experiment. On the same day the samples were used for GCMS analysis.

To evaluate the *Melissa officinalis* oil release, 1 mL of each liquid sample was added to 5 mL DCM and vortexed briefly before incubation overnight at 4 °C with shaking at 200 rpm to disperse the *Melissa officinalis* oil into DCM. The sample was centrifuged at 3000rpm (SIGMA 3-16 Centrifuge, Sigma Centrifuges, UK) for 5 min and the DCM, containing the essential oil, was collected in a 1 mL glass or plastic vial and analysed by GC-MS.

Controls included the contentment of the hydrogel prior incubation. To extract the oil within the hydrogel, 1 g of hydrogel was placed into 10mL DCM, vortexed briefly and incubated overnight at 4 °C with shaking at 200 rpm. After incubation, the suspension was centrifuged at 3000 rpm for 10 min and the DCM was collected in a vial and run in the GC-MS. All experiments were completed in triplicate and drug release was expressed as percentage cumulative release and milligrams released.

### 2.5. Candida albicans

*Candida albicans* 135BM2/94 was used to test the antifungal properties of the synthesised hydrogels. *Candida albicans* 135BM2/94 was a clinical strain from the School of Dentistry (Cardiff University, UK), which had been described as a high invader using an in vitro tissue model [21]. The strain was sub-cultured on Sabouraud Dextrose Agar (SDA) (Oxoid Ltd., UK) and grown at 37 °C for 24 h. A colony of *C. albicans* was inoculated into 20 mL of Sabouraud Dextrose Broth (SDB) (Oxoid Ltd., UK) and incubated aerobically with shaking (200 rpm) overnight at 37 °C.

### 2.6. Retention Assay

Overnight *Candida* cultures were washed and re-suspended to OD 1.0 at 600 nm, corresponding to concentrations of 3.7 × 10^4^ CFU/mL. Polymethylmethacrylate (PMMA) coupons (0.4 g), (0.1 cm^2^ × 0.4 cm) were fixed to the base of a sterilised container (70 cm^3^). Two replicate substrata were placed horizontally into the container and one of the 6 following preparations were added: 30 mL hydrogel containing 1% (*v*/*v*) *Melissa officinalis*; 30 mL hydrogel containing 2% (*v*/*v*) *Melissa officinalis*; 30 mL pure hydrogel without *Melissa officinalis*; 30 mL sterilised distilled water as a negative control; 30 mL of 2% (*v*/*v*) *Melissa officinalis* without the hydrogel; or 35 mL standardised *Candida* culture (positive control). The box with the hydrogel was placed on a heating plate (50 °C) for the hydrogel to jellify. The standardised *Candida* culture (35 mL of 3.7 × 10^4^ CFU/mL) was added and incubated for one hour at 37 °C aerobically.

Following incubation, the suspension was serial diluted and plated on SDA agar plates. The coupons were removed with sterile forceps, washed gently with 5 mL sterilised water and each coupon was transferred to a universal containing 2 mL phosphate buffered saline (PBS). The universals were vortexed (Hook and Tucker Rotamixer) for one minute and after that each coupon was added to a new universal with 2 mL PBS and again vortexed for one minute. The dilution of the first universal was added to the second universal, serial diluted and plated on SAD agar plates. To determine *Candida* growth in the hydrogel, 1–1.5 g of the hydrogel was weighed and diluted with 2 mL PBS, vortexed, serial diluted and plated on SDA plates. All concentrations were tested in quadruplicate and on three separate occasions. The method of Whitehead et al. [22] was used with minor adaptions (*n* = 3).

### 2.7. Disc Diffusion Method

The antifungal activity of 10% (*w*/*v*) methylcellulose hydrogels containing *Melissa officinalis* was evaluated. The method was adapted from that previously reported by Campos et al. (2014) [14]. Briefly, a 100 µL volume of an overnight *C. albicans* 135BM2/94 suspension was diluted to 10^7^ CFU/mL and uniformly spread onto SDA. The prepared hydrogel discs were placed onto the agar and incubated overnight at 37 °C, the diameter of zone of inhibition was measured (*n* = 9).

### 2.8. Time-Kill Assay

The method was adapted from that previously reported by Kong et al. (2016) [23]. One hundred and fifty µL of an overnight *C. albicans* 135BM2/94 culture diluted to 10^5^ CFU/mL was added to 150 µL of SDB in a sterile 1.5mL microcentrifuge tube. A disc of 0.75mm diameter (equivalent to 0.15 g) of 10% (*w*/*v*) methylcellulose hydrogels with 1% or 2% (*v*/*v*) *Melissa officinalis* essential oil was added to the tube. The cultures were incubated at 37 °C with shaking at 150rpm. At 2, 4, 6 and 24 h the suspension containing *C. albicans* 135BM2/94 was collected, and viable cell numbers counted. Briefly, samples were serially diluted in PBS and 50 µL were spread onto SDA plates using a Whitley Automated Spiral Plate system (WASP, Don Whitley Scientific Limited, UK). Agar plates were incubated overnight at 37 °C, and the resulting CFU/mL were counted. Controls included *C. albicans* cultured in SDB with or without 10% (*w*/*v*) methylcellulose hydrogel (*n* = 6).

### 2.9. Statistical Analysis

Statistical analysis was performed using GraphPad Prism Version 7.0 (GraphPad Software, La Jolla, CA, USA). Data were presented as arithmetic mean ± SD. The difference between treatments was statistically analyzed using one-way analysis of variance (ANOVA) followed by Tukey multiple comparisons test or T-test. Statistically significant differences were determined at *p* < 0.05.

## 3. Results

### 3.1. Optimisation of Hydrogel Containing Melissa Officinalis

**Figure 1:**
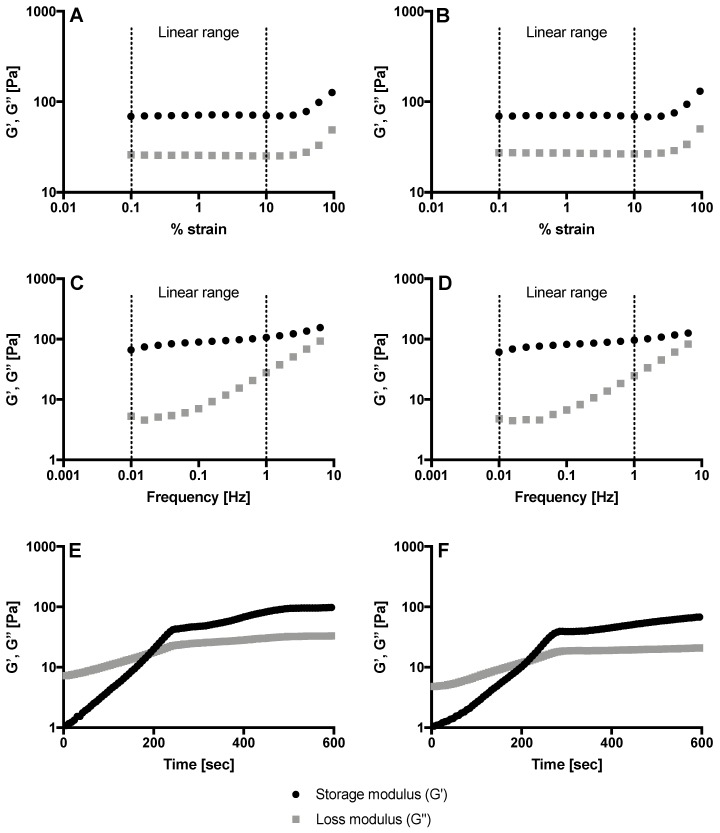
Rheological characterization of the synthesized methylcellulose hydrogels. (**A**,**B**) Strain sweep for 10% (*w*/*v*) methylcellulose hydrogel with 1% (*v*/*v*) *Melissa officinalis* (**A**) and 2% (*v*/*v*) *Melissa officinalis* (**B**). (**C**,**D**) Frequency sweep for 10% (*w*/*v*) methylcellulose hydrogel with 1% (*v*/*v*) *Melissa officinalis* (**C**) and 2% (*v*/*v*) *Melissa officinalis* (**D**). (**E**,**F**) Time sweep for 10% (*w*/*v*) methylcellulose hydrogel with 1% (*v*/*v*) *Melissa officinalis* (**E**) and 2% (*v*/*v*) *Melissa officinalis* (**F**) determined at an amplitude strain of 1% and angular frequency of 1 Hz. G′ (black circle) storage modulus and G″ (grey square) loss modulus.

The strain dependence of the storage (G′) and loss modulus (G″) of the hydrogel was measured with strain amplitude between 0.1% and 100% at a constant frequency of 1Hz. The storage modulus, which represents the hydrogel’s elasticity, was constant at strain amplitude below 10%, before increasing (>100 Pa), due to a re-arrangement of methylcellulose network and gel stiffening (Figure 1A,B). The frequency dependence of the storage and loss modulus was measured by stimulating the hydrogel with frequencies ranging from 0.01 Hz to 100 Hz at constant strain of 1%. At frequencies above 10 Hz, the loss modulus, which represents hydrogel’s viscous component, exceeded the storage modulus leading to gel disruption and loss of the hydrogels’ structure (Figure 1C,D). The gelation time was studied by observing G′ and G″ behaviour at 1% strain and 1Hz of frequency. In this range, the viscoelastic properties of the hydrogel were independent of the strain applied, as seen in Figure 1A,B,E,F show the storage and loss modulus as a function of time at a constant temperature of 37 °C. Initially, the solution was liquid (the loss modulus exceeded the storage modulus, G″ > G′), while over time the storage modulus increased reaching a time point called the ‘gelation time’ when storage and loss modulus intersected and the hydrogel was formed (G′ ≥ G″). Ten percent (10% (*w*/*v*)) methylcellulose hydrogel containing 1% (*v*/*v*) *Melissa officinalis* jellified in 167 s, whilst 10% (*w*/*v*) methylcellulose with 2% (*v*/*v*) *Melissa officinalis* jellified in 188 s. This difference was not significant (*p* = 0.054).

### 3.2. Melissa Officinalis Release

Prior to the evaluation of the release of the essential oil from the hydrogels, the composition of *Melissa officinalis* was analysed by GC-MS. Peaks were qualitatively identified by matching the mass spectra with those found in the NIST14 MS Search library. The quantification was carried out using a standard curve that correlated the peak areas to the concentrations. Three main compounds were identified, namely citronellal (50%), citronellol (10%) and geraniol (14%). In addition, 2.5% of linalool, a terpene with antimicrobial properties against oral pathogens, was also contained in the essential oil.

**Figure 2:**
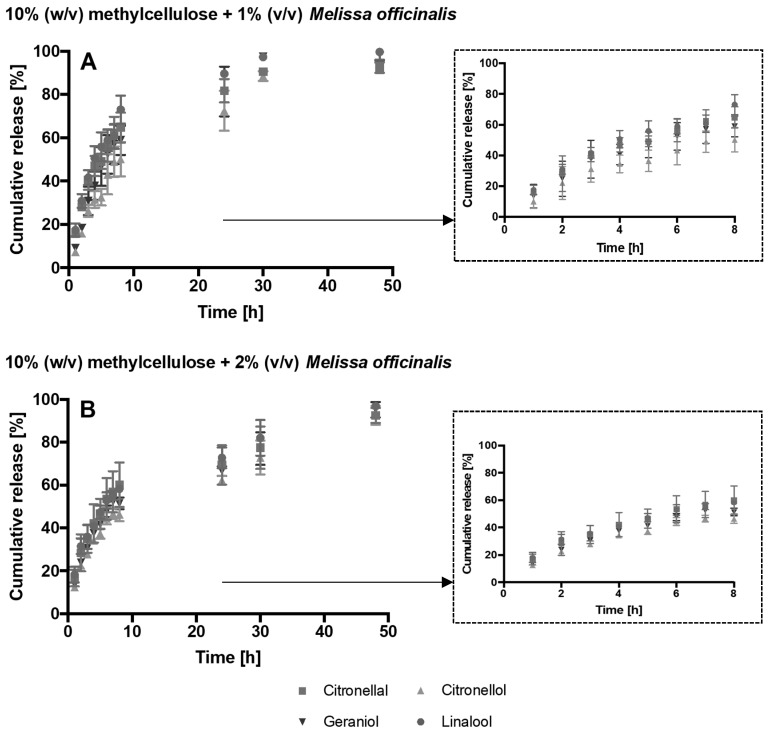
Percentage cumulative release of citronellal (square), citronellol (triangle), geraniol (inverted triange), and linalool (circle) of 10% (*w*/*v*) methylcellulose and 1% (*v*/*v*) *Melissa officinalis* (**A**), and 10% (*w*/*v*) methylcellulose and 2% (*v*/*v*) *Melissa officinalis* (**B**). A zoom of the percentage cumulative release between 0 and 8 h is also shown in the dashed rectangles.

**Figure 3:**
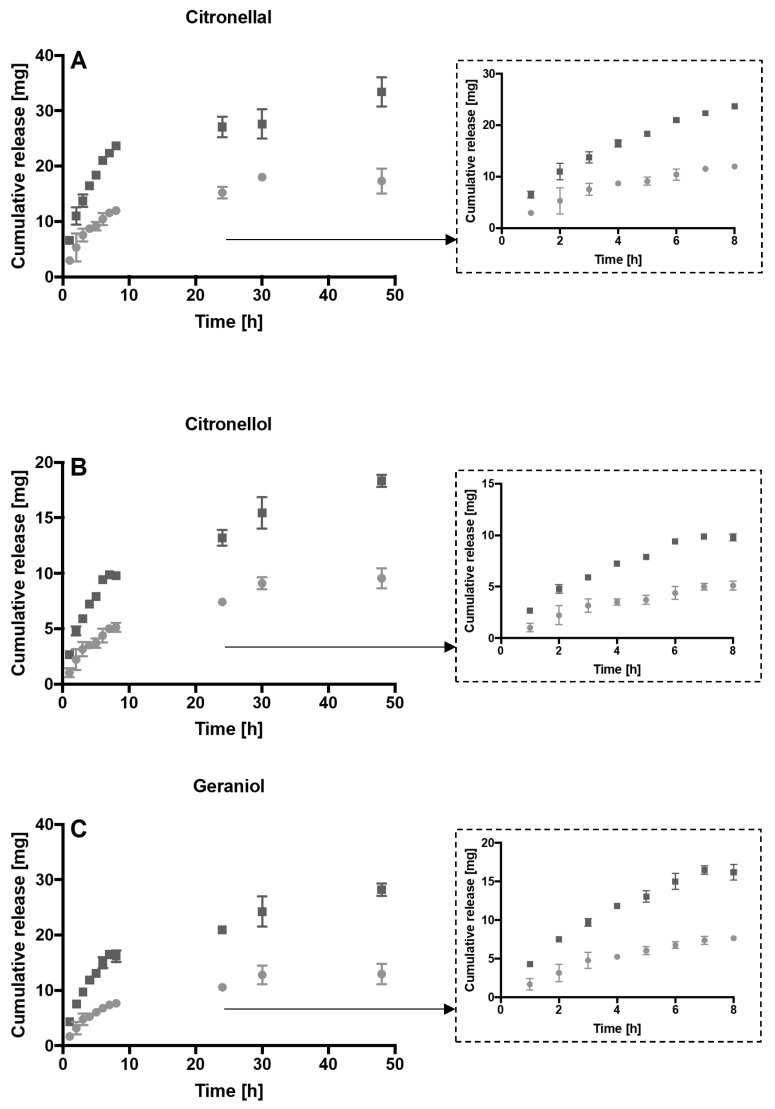

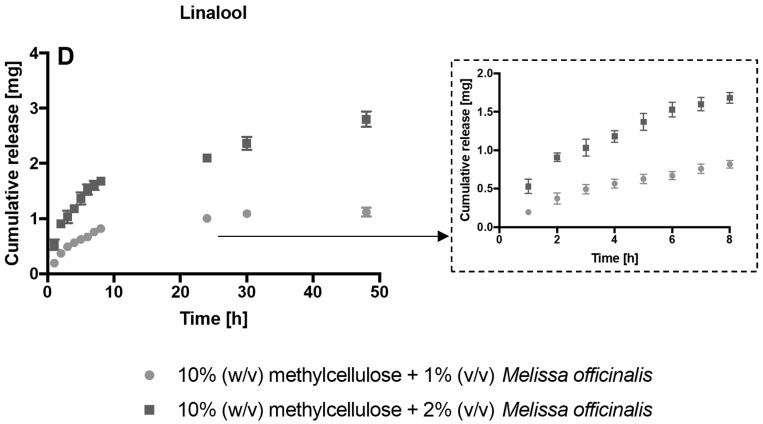
Cumulative release in milligrams of citronellal (**A**), citronellol (**B**), geraniol (**C**) and linalool (**D**) from 10% (*w*/*v*) methylcellulose with 1% (*v*/*v*) *Melissa officinalis* (circle), 10% (*w*/*v*) methylcellulose with 2% (*v*/*v*) *Melissa officinalis* (square). A zoom of the cumulative release between 0 and 8 h is also shown in the dashed rectangles.

Therefore, the release from the hydrogel of citronellol, citronellal, geraniol and linalool was investigated. The drug release profile from 10% (*w*/*v*) methylcellulose hydrogels with 1 or 2% (*v*/*v*) *Melissa officinalis* essential oil over 48 h was demonstrated (Figure 2). Release was maintained for the duration of the experiment. After 8 h more than the 45% of the compounds was released, while within 48 h more than the 92% of each compound leached out. As expected, by increasing the initial concentration of *Melissa officinalis* into the hydrogel, the amount of drug release increased (Figure 3).

### 3.3. Retention Assay

**Figure 4:**
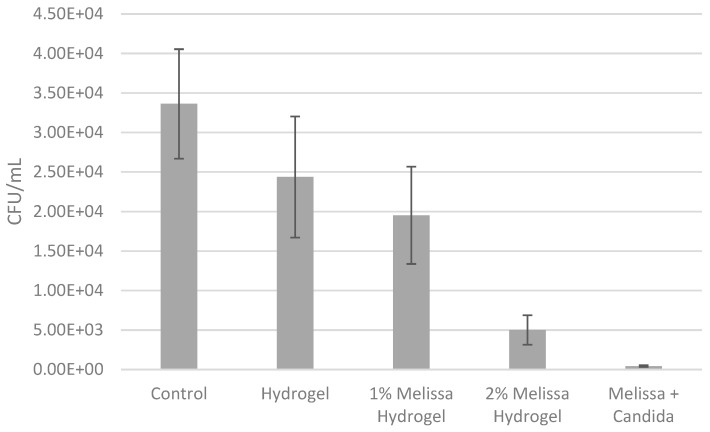
Retention assay after 1 h of exposure of *C. albicans* to 10% (*w*/*v*) methylcellulose hydrogels with or without 1 or 2% (*v*/*v*) *Melissa officinalis*.

The 10% (*w*/*v*) methylcellulose hydrogels with 2% (*v*/*v*) *Melissa officinalis* essential oil significantly reduced the retention of *C. albicans* (5.01 × 10^3^ CFU/mL; *p* = 0.03). There was also a significant difference in the reduction of the *C. albicans* retained on the hydrogels when loaded with either a 1% or 2% Melissa oil (*p* = 0.003). The lowest retention was achieved with 2% (*v*/*v*) *Melissa officinalis* on its own (solution), without the addition of the hydrogel (4.31 × 10 ^2^ CFU/mL) (Figure 4).

### 3.4. Disk Diffusion Method

The antifungal activity of the methylcellulose hydrogels was determined by the disc diffusion method and the inhibitory zone measured (Table 1). The 10% (*w*/*v*) methylcellulose hydrogels with 1 or 2% (*v*/*v*) *Melissa officinalis* essential oil exhibited antifungal activity (*p* < 0.0001), while methylcellulose itself was not antifungal.

### 3.5. Time-Kill Assay

**Figure 5:**
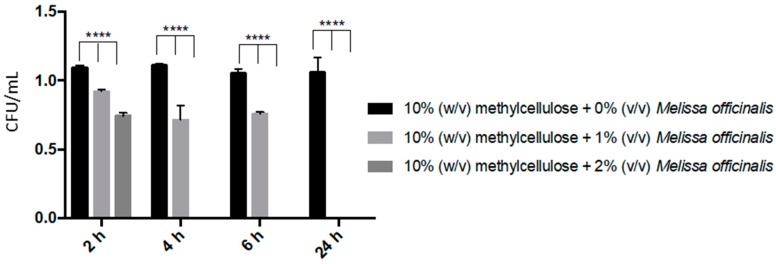
In vitro time-kill assay after 2, 4, 6 and 24 h of exposure of *C. albicans* to 10% (*w*/*v*) methylcellulose hydrogels with or without 1 or 2% (*v*/*v*) *Melissa officinalis*. **** = *p* < 0.0001.

Antifungal activity was evaluated with a time-kill assay. The amount of *C. albicans* recovered within 2 h was decreased significantly (*p* < 0.001) by 15% and 30% in the presence of 10% (*w*/*v*) methylcellulose hydrogel with 1% (*v*/*v*) *Melissa officinalis* and 10% (*w*/*v*) methylcellulose hydrogel with 2% (*v*/*v*) *Melissa officinalis,* respectively (Figure 5). After 4 h application of 10% (*w*/*v*) methylcellulose hydrogels with 2% (*v*/*v*) *Melissa officinalis*, no viable cells were recovered, while it took 24 h to completely kill *C. albicans* when it was cultured in the presence of 10% (*w*/*v*) methylcellulose hydrogels with 1% (*v*/*v*) *Melissa officinalis* (Figure 5). It was observed that the presence of 10% (*w*/*v*) methylcellulose promoted *C. albicans* growth. This difference was significant until 6h incubation (*p* < 0.003), but not after 24 h incubation (*p* > 0.33).

## 4. Discussion

*Candida* species are commensal microorganisms of the oral cavity, mainly found on the posterior part of the tongue and on the oral mucosa. *Candida* is an opportunistic pathogen and can cause oral candidiasis when suitable conditions arise.

The emergence of antimicrobial resistance, defined by the World Health Organization (WHO) as a global threat to the effective treatment of infections, has led to a new interest in the antifungal properties of natural and chemical compounds [24]. *Melissa officinalis* has been previously reported to be active against *C. albicans* both in the planktonic and biofilm form at Minimum Inhibitory Concentrations (MIC) of 0.06% (*v*/*v*) and 1.5% (*v*/*v*), respectively [11]. *Candida albicans* is a predominately studied yeast because it significantly changes its morphology and expresses a range of virulence factors associated with biofilm formation, invasion of the host tissue and adaptation to the environment [25,26,27,28]. Being lipophilic, essential oils typically integrate into membrane structures causing increased cell permeability, leaching of intracellular components and inactivation of enzymes [5,29]; blocking the synthesis of membrane; inhibiting spore germination, proliferation of fungi and cellular respiration [28,30]. Furthermore, essential oils can also interact with the mitochondrial membrane leading to cidal effects [4]. Therefore, a hydrogel to be used as delivery vehicle to release the essential oil at the site of infection was developed. It is important to emphasize that methylcellulose is an US Food and Drug Administration (FDA)-approved material, it is approved for food industry and biomedical applications and it has been tested over the years to be not toxic for human and animal use [31,32,33].

Ten percent (10% (*w*/*v*)) methylcellulose hydrogels with 1 or 2% (*v*/*v*) *Melissa officinalis* essential oil were successfully synthesised. Rheological analysis was performed to evaluate the gelation time at body temperature. The essential oil content did not influence the rheological properties, and no significant differences in the loss and storage modulus were observed. The synthesised hydrogels are intended to be used as vehicles for drug delivery. Accordingly, it was important that once applied into the mouth, they undergo gelation at 37 °C within an appropriate time. For this reason, the viscoelastic properties of the hydrogel were evaluated to investigate the gelation time in the linear viscoelastic region at body temperature. It should be noted that these hydrogels were not supposed to be used to resist deformations such as in load-bearing applications (e.g., cartilage and bone regeneration). Considering the gelation time, the hydrogels were considered suitable for an oral application, being able to form a self-supporting hydrogel at 37 °C in less than three minutes.

Before investigating the essential oil release from the hydrogels, the composition of *Melissa officinalis* essential oil was analysed by GC-MS, since it is known that the composition and the ratio of the components depend on the plant’s origins and influence the antimicrobial properties of the essential oils [5]. The main component of the oil was citronellol (50% (*v*/*v*)) followed by geraniol (14% (*v*/*v*)) and citronellal (10% (*v*/*v*)). Two-point five percent (2.5% (*v*/*v*)) of linalool, a terpene with antimicrobial properties and generally recognized as safe by the FDA [34] was also contained in *Melissa officinalis* essential oil. Therefore, it was decided to evaluate the release of the three main compounds as well as of linalool.

Hydrogels are characterised by a 3D-polymeric network that allows liquid and molecules to diffuse. The diffusion of the drug depends on the mesh size: if the mesh size is larger than the drug then molecules are free to migrate through the network, while if the drug size is comparable to the mesh size then the drug is physically entrapped inside the hydrogel [35]. However, the mesh size can change over time allowing the release of the drug. Two main phenomena can lead to changes in the 3D-polymeric network, namely, degradation and swelling. Degradation, a loss of polymer mass, can be mediated by enzyme activity or by hydrolysis. Degradation results in an increase of mesh size that allows drug diffusion. Similarly, swelling due to water absorption, increases the mesh size and allows drug release [35]. In general, diffusion and drug release are affected by different parameters such as the hydrophilicity/hydrophobicity of the drug, the surface area and geometry of the hydrogel, the type and volume of solvent, the degradation of the hydrogel, the mesh size and the swelling rate [36]. Different volumes, solvents, ratios of hydrogel to solvent, flasks (e.g., universal, conical flasks), the presence of dialysis membranes and different shaking rate were found to be variable in a review of the literature [23,37,38,39,40]. In the current study, the drug release was evaluated over 48 h, the amount of compounds released were a function of the initial content of oil. Indeed, the amount released from the hydrogel containing 2% (*v*/*v*) of *Melissa officinalis* essential oil was significantly greater than that released from the hydrogels synthesized with 1% (*v*/*v*) of *Melissa officinalis* essential oil.

The antifungal activity of the hydrogels was screened against a clinical strain described previously as a high tissue invader (*C. albicans* 135BM2/94). The clinical strain had previously been isolated from a patient with chronic hyperplastic candidiasis and squamous cell carcinoma [21]. *Candida albicans* was not recovered when cultured in the presence of (10% (*w*/*v*) methylcellulose with 2% (*v*/*v*) *Melissa officinalis* essential oil) for 4 h with (10% (*w*/*v*) methylcellulose with 1% (*v*/*v*) *Melissa officinalis* essential oil for 24 h. Besides evaluating the antifungal potential of the drug released from the hydrogel, the antifungal potential of methylcellulose itself was investigated by comparing the viable cell count with that obtained culturing *C. albicans* in broth without the hydrogel.

The disc diffusion method demonstrated that the methylcellulose on its own did not inhibit *Candida*. In contrast, when the methylcellulose hydrogel contained *Melissa officinalis* essential oil, a zone of inhibition was observed and it was proportional to the amount of *Melissa officinalis* contained in the hydrogel. These results were in accordance with those of Campos et al. [14] and Ayana and Turhan [15] who observed a zone of inhibition when methylcellulose hydrogel was loaded with Ginja cherry or olive oil extracts, respectively.

The retention assay was used to observe if the methylcellulose hydrogel could be used as a direct drug delivery system to inhibit the retention of *C. albicans* on PMMA denture surfaces. For this reason, the hydrogel suitability for antimicrobial applications was tested on custom-made PMMA coupons. The 10% (*w*/*v*) methylcellulose hydrogels with 2% (*v*/*v*) *Melissa officinalis* essential oil significantly reduced the retention of *C. albicans* compared to the hydrogel on its own and to the 1% (*v*/*v*) *Melissa officinalis* hydrogel on PMMA. It is important to highlight that the lowest retention was achieved with 2% (*v*/*v*) *Melissa officinalis* pure oil without the application of the hydrogel. However, it is worth noting that in an in vivo situation firstly the 2% (*v*/*v*) essential oil concentration would not be directed to the target but, due to its liquid form, it would spread around on the surrounding tissues. Secondly, the 2% (*v*/*v*) concentration would be quickly diluted in the oral cavity, diminishing its effective antimicrobial potency. Finally, having the essential oil in its liquid form would make it difficult to determine what drug concentration will reach the target area. Other studies have focused on hydrogels as delivery systems for the oral cavity [41,42,43]. Kong et al. (2016) developed a hydroxypropyl methylcellulose hydrogel for use against oral candidiasis. A 4% (*w*/*w*) hydroxypropyl methylcellulose hydrogel was formulated with 2 mg/mL of Histatin-5, a peptide with antimicrobial properties. The authors tested the antifungal activity of the hydrogel against *C. albicans* standard strain SC5314, observing Histatin-5 release from the hydrogel within 2 h and a significant decrease in CFU/mL compared to the control (i.e., hydroxypropyl methylcellulose hydrogel without antimicrobial). In addition, the novel formulation was tested in vivo. The tongues of mice were infected with *C. albicans* and the hydrogel was applied for 1 h. Untreated mice developed oral candidiasis with tissue damage and *C. albicans* was recovered from the site. By contrast, most of the mice treated with the hydrogel showed less fungal adherence, hyphal penetration and tissue damage [23].

## 5. Conclusions

A 10% (*w*/*v*) methylcellulose hydrogel with 1% (*v*/*v*) and 2% (*v*/*v*) *Melissa officinalis* was successfully synthesised. The rheological analysis highlighted that the hydrogels jellified at body temperature in less than 3 min. The antifungal properties of the hydrogels were confirmed by both zone of inhibition method and time-kill assays and the 2% (*v*/*v*) hydrogel significantly reduced the retention of *C. albicans*. The novel findings of this work in future, will be expanded to include a range of Candida species as well as bacterial species associated with oral infections.

## Figures and Tables

**Table 1:** Inhibitory zone diameters of 10% (*w*/*v*) methylcellulose hydrogels with or without 1 or 2% (*v*/*v*) *Melissa officinalis*. **** = *p* < 0.0001.

	Inhibitory Zone Diameter (mm)
10% (*w*/*v*) methylcellulose	0
10% (*w*/*v*) methylcellulose + 1% (*v*/*v*) *Melissa officinalis*	10.2 ± 0.4
10% (*w*/*v*) methylcellulose + 2% (*v*/*v*) *Melissa officinalis*	17.5 ± 2.6 (****)
